# A Tertiary Centre’s Experience With Using Ranibizumab Biosimilar Compared to Aflibercept for Neovascular Age-Related Macular Degeneration: A Retrospective Study

**DOI:** 10.7759/cureus.75586

**Published:** 2024-12-12

**Authors:** Ahmed Bilal, Muslim Bilal, Danyal Usman, Aarij Elahi, Ayad Al-Bermani

**Affiliations:** 1 Ophthalmology, University Hospital of Wales, Cardiff, GBR; 2 School of Medicine, Cardiff University, Cardiff, GBR; 3 Internal Medicine, University Hospital of Wales, Cardiff, GBR; 4 Ophthalmology, Royal Glamorgan Hospital, Llantrisant, GBR

**Keywords:** aflibercept, anti-vascular endothelial growth factor, biosimilar, eylea, intravitreal anti-vegf, neovascular age-related macular degeneration, ongavia, ranibizumab biosimilar, real-world data, retina

## Abstract

Objective

This study aims to evaluate the real-world efficacy of ranibizumab biosimilar (Ongavia), compared to aflibercept (Eylea), in the treatment of treatment-naïve neovascular age-related macular degeneration (nAMD) at a busy tertiary eye care centre.

Methods

A retrospective analysis of medical records from August 2022 to August 2024 was conducted, comparing treatment outcomes in treatment-naive nAMD patients who received either Ongavia or Eylea intravitreal anti-VEGF (vascular endothelial growth factor) injections under a treat-and-extend protocol. Initial and 12-month outcome measures post-treatment initiation were collected, including best-corrected visual acuity (BCVA), central retinal thickness (CRT), prescribed treatment intervals, actual injection frequency, and the average total number of injections per eye over 12 months.

Results

A total of 62 eyes met the inclusion criteria. Over 12 months of follow-up, patients receiving Eylea (n = 36) showed a significantly greater improvement in BCVA (7.08 ± 4.12), p = 0.018, compared to Ongavia (n = 26) (-1.9 ± 3.31). CRT reductions were also more substantial for Eylea (-116.21 µm ± 35.61 µm) than for Ongavia (-51.14 µm ± 22.21 µm), p = 0.002. The average number of injections was 6.55 for Ongavia and 5.75 for Eylea over the 12-month follow-up. Excluding the initial three loading doses, observed injection intervals averaged 9.49 weeks for Eylea and 8.17 weeks for Ongavia. Notably, for the total study period, 164 out of 171 (96%) Ongavia injections were prescribed at four-week intervals, compared to 110 out of 207 (53%) for Eylea. However, capacity constraints impacted adherence to prescribed dosing schedules, affecting efficacy.

Conclusion

Our study indicates reduced visual and morphological outcomes with Ongavia compared to Eylea in treating nAMD when monthly injections cannot be provided as prescribed. Given clinical capacity constraints, Eylea’s greater potency and durability prove advantageous, allowing extended intervals and reducing the reliance on strict monthly dosing. This highlights the need for additional resources to support frequent biosimilar administration and ensure effective ranibizumab treatment. A comprehensive cost-benefit analysis is warranted to assess whether increased clinic capacity offsets Ongavia's lower per-injection cost, compared to Eylea.

## Introduction

Age-related macular degeneration (AMD) is the leading cause of severe visual impairment and blindness among individuals aged 50 years and older in developed nations, accounting for over 8.7% of global blindness [1,2]. It is classified into two primary forms: dry (atrophic) AMD, which is more common but progresses gradually, and wet (neovascular) AMD (nAMD), characterised by choroidal neovascularisation (CNV), leading to rapid vision loss if untreated. In nAMD, abnormal blood vessels grow under the retinal pigment epithelium and into the macula, causing fluid leakage, haemorrhage, and fibrosis, ultimately impairing central vision [3].

The macula, located at the central part of the retina, encompasses the fovea, which is specialised for high-acuity vision and essential for tasks such as reading and recognising faces. The anatomical and functional integrity of the macula is critical for maintaining visual function. Disruption of the macula due to fluid accumulation and neovascularisation in nAMD can result in severe central vision loss, leaving peripheral vision relatively unaffected [4].

At a molecular level, vascular endothelial growth factor (VEGF) plays a central role in the pathogenesis of nAMD. VEGF is a signalling protein that promotes angiogenesis and increases vascular permeability. Its upregulation in response to retinal hypoxia contributes to the development of pathologic neovascularisation in nAMD [5]. Anti-VEGF therapies have become the cornerstone of nAMD treatment, targeting VEGF to prevent angiogenesis and fluid leakage, thereby preserving macular function and vision.

Ranibizumab (Lucentis) and aflibercept (Eylea) are two widely used anti-VEGF agents. Ranibizumab, a humanised monoclonal antibody fragment targeting VEGF-A, was approved by the U.S. Food and Drug Administration (FDA) in 2006. Its smaller molecular structure allows for high binding affinity but necessitates frequent dosing due to its shorter intraocular half-life [6]. Aflibercept, a fusion protein approved in 2011, binds not only VEGF-A but also VEGF-B and placental growth factor (PlGF), providing broader inhibition and longer durability, allowing for extended treatment intervals in many patients [7].

These anti-VEGF agents are typically administered via intravitreal injections, with dosing schedules tailored to disease activity. The treat-and-extend protocol, widely adopted in clinical practice, involves initial monthly loading doses followed by interval extension based on treatment response [8]. While both ranibizumab and aflibercept have demonstrated significant efficacy in preserving and improving vision in controlled clinical trials, real-world implementation often faces challenges such as capacity constraints, treatment adherence, and variability in treatment intervals, which can impact outcomes [9].

The expiration of ranibizumab’s patent in June 2020 has allowed the development of biosimilars, which are biologic drugs designed to replicate the reference molecule in structure, function, efficacy, and safety. Ranibizumab biosimilars, including Ongavia, were approved in the UK as cost-saving alternatives to Lucentis. Ongavia, priced significantly lower than the originator molecule, offers an economically attractive option for healthcare systems under financial strain. While ranibizumab biosimilars are designed to replicate the pharmacologic efficacy and safety of innovator ranibizumab (Lucentis), limited data exist comparing their real-world efficacy to the well-known standard of care, aflibercept (Eylea), especially when prescribed treatment intervals cannot be adhered to [10,11].

This study evaluates the real-world efficacy of the ranibizumab biosimilar Ongavia compared to aflibercept in a capacity-constrained tertiary centre, when delays in treatment intervals occurred due to service capacity strain. By analysing functional (best-corrected visual acuity, BCVA) and anatomical (central retinal thickness, CRT) outcomes, as well as injection intervals and frequency, we aim to provide insights into the comparative performance of these anti-VEGF agents under real-world conditions, addressing both clinical efficacy and resource implications. This includes assessing how Ongavia and Eylea treatments performed under unplanned appointment delays that occurred at our centre.

## Materials and methods

Study design and setting

This single-centre retrospective audit was conducted at a tertiary hospital ophthalmology medical retina unit to evaluate the use of two types of intravitreal anti-VEGF injections initiated for new presentations of nAMD to the medical retina department. The study assessed 12-month post-initial intravitreal injection outcome data from August 2022, the roll-out date of the ranibizumab biosimilar Ongavia at our centre, to August 2024. Medical records for treatment-naïve nAMD patients treated with Ongavia or Eylea intravitreal injections under a treat-and-extend protocol were reviewed. For both Ongavia and Eylea, the treat-and-extend protocol consisted of three initial four-weekly injections, followed by an extension of the injection interval dependent on disease progression, as per Royal College of Ophthalmologists guidance [8]. No intervals were less than four weeks.

Selection criteria

The hospital policy and treatment pathway involved using Ongavia as the first-line treatment for treatment-naive nAMD. Therefore, the decision to initiate Ongavia or Eylea for any individual eye was based on the choice of the treating clinician, who could start either of the two intravitreal injections.

Inclusion and exclusion criteria

As a cost-saving measure, there was a major intravitreal injection switch initiative at our centre to switch all patients who were receiving the more expensive ranibizumab innovator, Lucentis, to the cheaper alternative, ranibizumab biosimilar Ongavia, beginning in August 2022. However, only treatment-naïve patients initiating Ongavia or Eylea therapy within the study period were included. Exclusions included a history of prior anti-VEGF therapy, incomplete loading phases (fewer than three injections), and eyes with other ocular pathology or interventions that could confound visual acuity outcomes.

Outcome measures

Primary outcomes included changes in BCVA, recorded as ETDRS (Early Treatment Diabetic Retinopathy Study) letters, and CRT. Secondary outcomes assessed prescribed injection treatment intervals, actual injection frequency, and total number of injections.

Statistical analysis

To assess statistical significance, a paired samples t-test was performed for comparison between the same continuous variables: initial and endpoint BCVA, and initial and endpoint CRT. Statistical significance for all tests was set at a threshold of p < 0.05. IBM SPSS Statistics for Windows, Version 27 (Released 2020; IBM Corp., Armonk, NY, USA) was used to compile all data and conduct statistical paired samples t-test analysis between results for initial and endpoint BCVA and CRT.

Ethics approval

This study is a retrospective audit of two intravitreal injections, licensed by the institution's pharmacy and used in accordance with hospital guidelines. Relevant institutional approval has been obtained, and no patient-identifiable data are included. As this audit is not classified as research by the IRB and involves fully anonymized data, no IRB approval is required. The audit was granted an exemption from IRB review by the Clinical Governance and Audit Department of Cardiff & Vale University Health Board, NHS Trust.

## Results

**Figure 1:**
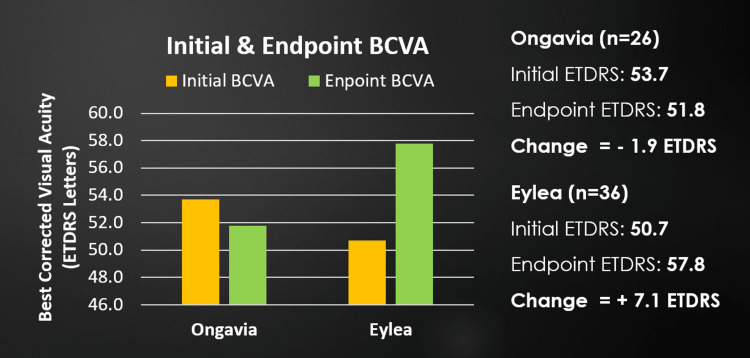
Average initial and endpoint BCVA for Ongavia and Eylea. BCVA, Best-corrected visual acuity; ETDRS, Early treatment diabetic retinopathy study

A total of 62 eyes (34 female and 28 male) met the inclusion criteria. Patients receiving Eylea (n = 36) showed a significantly greater improvement in BCVA ETDRS letters of 7.08 ± 4.12, compared to Ongavia (n = 26), with -1.9 ± 3.31, p = 0.018. The mean initial and endpoint BCVA for Eylea was 50.7 and 57.8 ETDRS letters, respectively. The mean initial and endpoint BCVA for Ongavia was 53.7 and 51.8 ETDRS letters, respectively (Figure 1). 

**Figure 2:**
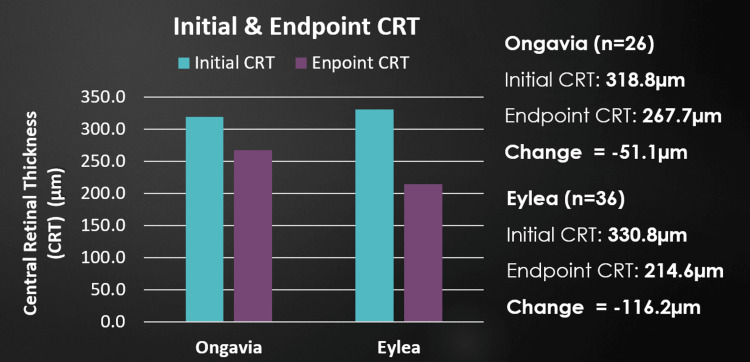
Average initial and endpoint CRT (µm) for Ongavia and Eylea. CRT, Central retinal thickness

CRT reductions were greater in the Eylea group (-116.21 µm ± 35.61 µm), versus -51.14 µm ± 22.21 µm for Ongavia, p = 0.002. The mean initial and endpoint CRT for Eylea was 330.8 µm and 214.6 µm, respectively. The mean initial and endpoint CRT for Ongavia was 318.8 µm and 267.7 µm, respectively (Figure 2).

**Figure 3:**
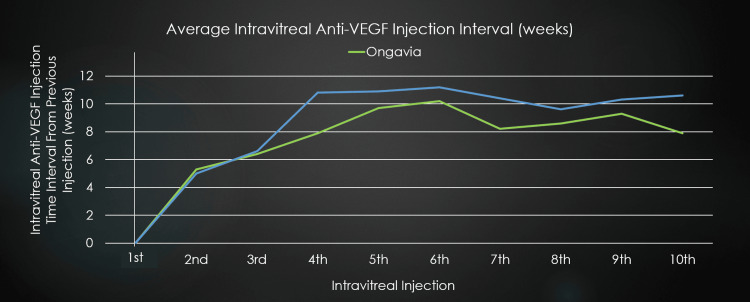
Average time interval (weeks) since previous intravitreal injection for Ongavia and Eylea. VEGF, Vascular endothelial growth factor

Excluding the initial three monthly loading doses for both anti-VEGF injections, the average observed injection intervals were 9.49 weeks for Eylea, compared to 8.17 weeks for Ongavia (Figure 3). Notably, for the total study period, 164 out of 171 (96%) Ongavia injections were prescribed at four-week intervals, compared to 110 out of 207 (53%) for Eylea, which were prescribed at four-week intervals post-loading phases. Nevertheless, capacity limitations prevented strict adherence to monthly dosing intervals due to clinical capacity constraints, causing significant injection appointment delays. The first three intravitreal injections were pre-booked for four-week intervals; however, the second injection was administered on average at 5.3 and 5 weeks for Ongavia and Eylea, respectively. Likewise, the third injection was administered on average at 6.4 and 6.6 weeks for Ongavia and Eylea, respectively.

## Discussion

Studies that are conducted under controlled environments with consistent treatment intervals, therefore allowing optimal usage of ranibizumab biosimilar, show good visual and morphological improvements [12]. Likewise, several studies of ranibizumab biosimilar have shown non-inferiority when compared to the original innovator ranibizumab (Lucentis) [13,14]. However, the real-world capacity constraints of over-prescribed clinics are not the same as the controlled study environment, hence the importance of real-world data to assess the treatment efficacy of anti-VEGF intravitreal injections outside of controlled trial environments, where the rising demand for ophthalmology care is outstretching capacity to provide eye care in the UK [15,16]. 

Biochemical and molecular differences

Ranibizumab (and its biosimilar Ongavia) and aflibercept target VEGF to reduce neovascularisation and fluid leakage in nAMD. However, they differ structurally and mechanistically, influencing their pharmacodynamics and therapeutic efficacy. Ranibizumab is a smaller Fab (fragment antigen-binding) fragment, selectively inhibiting VEGF-A isoforms with a high binding affinity yet a shorter half-life, typically necessitating monthly dosing to maintain therapeutic levels [17]. Conversely, aflibercept is a larger fusion protein that binds VEGF-A, VEGF-B, and PlGF, offering broader inhibition and an extended intraocular duration due to its full-length structure, which allows effective use in treat-and-extend protocols [18].

These structural differences underscore aflibercept’s durability advantage, providing sustained suppression of VEGF activity - particularly valuable in patients requiring extended intervals due to capacity limitations [19]. This biochemical profile renders aflibercept favourable in clinical settings where patient volumes may delay consistent monthly administration as prescribed by ophthalmologists.

Impact of capacity constraints

In real-world practice, capacity constraints can significantly hinder the timely administration of anti-VEGF injections - a challenge exacerbated by the high patient volume and limited intravitreal injection slots at many tertiary centres [20]. In a clinical trial, Ongavia administered injections at four-week intervals, achieving comparable efficacy to Lucentis under these controlled conditions [21]. However, replicating this schedule in real-world settings with limited resources presents substantial logistical challenges. Studies have demonstrated that extended intervals beyond four weeks for ranibizumab are associated with reduced efficacy and increased disease recurrence compared to aflibercept [9,22,23]. These findings align with our observations, where capacity constraints resulted in unplanned extended intervals for Ongavia, contributing to its comparatively lower efficacy in BCVA and CRT outcomes.

Aflibercept’s longer duration of action allows greater flexibility in dosing intervals, reducing the risk of vision decline associated with delayed appointments. In contrast, patients on Ongavia, subject to more frequent dosing requirements, may face suboptimal outcomes when injection intervals are prolonged due to oversubscribed clinic schedules [24]. This discrepancy highlights the need for healthcare systems to adapt infrastructure and appointment availability to meet the specific dosing needs of cheaper-cost, yet more resource-delivery-dependent, biosimilars like Ongavia.

An increased number of intravitreal injections is also linked to an increased theoretical chance of adverse effects, some of which can be devastating to vision, such as endophthalmitis. Therefore, utilising a more potent and durable anti-VEGF treatment is potentially safer, less intrusive, and potentially cost-saving overall for ophthalmology services [25]. The volume of extra workload placed on an already oversubscribed ophthalmology service by needing more frequent injections further confounds pressure on waiting lists and appointment availability [26].

Clinician bias and patient selection

A potential bias was observed, with clinicians more likely to select aflibercept for patients presenting with perceivably worse and more advanced disease. This selection bias will, of course, affect the visual gains data by possibly allowing for greater recoverable visual and morphological potential for the Eylea cohort. However, this does not explain the significantly lower endpoint BCVA and reduced reduction of endpoint CRT observed in the Ongavia cohort compared to the Eylea cohort. Clinicians may perceive Eylea to be more effective in patients requiring robust VEGF inhibition due to its proven long track record and clinically observed superiority over ranibizumab at our institution, which was the initiating question leading to this study.

This bias to initiate those with worse disease states on Eylea potentially also affected the ability to further increase injection intervals for the Eylea cohort, as more advanced diseases often require more frequent injections. Even with all the above in mind, the Eylea cohort received less frequent injections with longer injection intervals overall, had patients with worse average initial BCVA and CRT compared to the Ongavia cohort, and still performed significantly better than Ongavia (Figures 1-3).

Additionally, logistical challenges, such as Ongavia’s preparation requirements (e.g., vial handling, drawing up medication with needle and syringe, and potential medication residue), introduce variability in administration, with possible impacts on therapeutic outcomes and patient satisfaction [27]. This is in contrast to Eylea’s manufacturer-packaged pre-filled syringe delivery system, which requires minimal preparation and no manual preparation of medication [28].

Ending discussion statement 

In a real-world setting, where timely care is critical, the question becomes: would you choose Ongavia for a loved one if Eylea is an option? Our findings underscore the importance of prioritising efficacy over cost in capacity-limited environments, advocating for treatments that reliably deliver the best outcomes when every appointment counts.

Additional observations

Notably, both cohorts experienced unwanted extended injection intervals, even during loading phases, due to limitations in appointment availability and service capacity strain, impacting the effective prescribed and intended delivery of intravitreal injections. This study provides insight into how Eylea and Ongavia performed under such service strains and delayed appointments at our centre. There were multiple consultant ophthalmologists managing the patients involved in this cohort, which can lead to variations in the initiation and prescription of injection intervals. The study cohorts are small, and a larger number of patients may provide a more conclusive correlation.

## Conclusions

This retrospective study demonstrates that, under real-world conditions, ranibizumab biosimilar (Ongavia) exhibits reduced efficacy in functional and morphological outcomes compared to aflibercept (Eylea) for nAMD treatment. Eylea’s superior potency and durability provide critical advantages, particularly in resource-constrained clinical settings, where strict monthly injection schedules are challenging to maintain.

Given the increasing demand for ophthalmology services, and how common appointment delays can be, our findings support the preferential use of Eylea compared to Ongavia in capacity-limited settings to ensure optimal visual outcomes. Future studies should perform a thorough cost analysis to investigate the long-term cost implications and clinical efficacy of biosimilars under various administrative constraints to help guide their implementation.

## References

[1] Li JQ, Welchowski T, Schmid M, Mauschitz MM, Holz FG, Finger RP (2020). Prevalence and incidence of age-related macular degeneration in Europe: a systematic review and meta-analysis. Br J Ophthalmol.

[2] Mitchell P, Liew G, Gopinath B (2018). Age-related macular degeneration. Lancet.

[3] Nguyen V, Barthelmes D, Gillies MC (2021). Neovascular age-related macular degeneration: a review of findings from the real-world Fight Retinal Blindness! registry. Clin Exp Ophthalmol.

[4] Ptito M, Bleau M, Bouskila J (2021). The retina: a window into the brain. Cells.

[5] Chandra S, Arpa C, Menon D (2020). Ten-year outcomes of antivascular endothelial growth factor therapy in neovascular age-related macular degeneration. Eye (Lond).

[6] Rosenfeld PJ, Brown DM, Heier JS, Boyer DS, Kaiser PK, Chung CY, Kim RY (2006). Ranibizumab for neovascular age-related macular degeneration. N Engl J Med.

[7] Heier JS, Bressler NM, Avery RL (2016). Comparison of aflibercept, bevacizumab, and ranibizumab for treatment of diabetic macular edema: extrapolation of data to clinical practice. JAMA Ophthalmol.

[8] Ross AH, Downey L, Devonport H (2020). Recommendations by a UK expert panel on an aflibercept treat-and-extend pathway for the treatment of neovascular age-related macular degeneration. Eye (Lond).

[9] Holz FG, Tadayoni R, Beatty S (2015). Multi-country real-life experience of anti-vascular endothelial growth factor therapy for wet age-related macular degeneration. Br J Ophthalmol.

[10] Hatamnejad A, Dadak R, Orr S, Wykoff C, Choudhry N (2023). Systematic review of efficacy and meta-analysis of safety of ranibizumab biosimilars relative to reference ranibizumab anti-VEGF therapy for nAMD treatment. BMJ Open Ophthalmol.

[11] Sharma A, Kondo M, Iwahashi C (2023). Approved biosimilar ranibizumab - a global update. Eye (Lond).

[12] Ghosh AK, Nikumbh US, Shukla CK (2024). Efficacy, safety and immunogenicity of sun’s ranibizumab biosimilar in neovascular age-related macular degeneration: a phase 3, double-blind comparative study. Ophthalmol Ther.

[13] Rana PJ, Deshmukh H, Shah U (2024). Efficacy and safety of biosimilar ranibizumab (OPTIMAB®) versus innovator ranibizumab in patients with neovascular (wet) age-related macular degeneration: a double-blind, randomized, multicenter, phase III study. Clin Ophthalmol.

[14] Chakraborty D, Mondal S, Boral S (2023). Biosimilar versus innovator molecule of ranibizumab in neovascular age-related macular degeneration (the balance trial): real-world evidence. Clin Ophthalmol.

[15] Buchan JC, Amoaku W, Barnes B (2017). How to defuse a demographic time bomb: the way forward?. Eye (Lond).

[16] Buchan JC, Norman P, Shickle D, Cassels-Brown A, MacEwen C (2019). Failing to plan and planning to fail. Can we predict the future growth of demand on UK Eye Care Services?. Eye (Lond).

[17] Frampton JE (2013). Ranibizumab: a review of its use in the treatment of neovascular age-related macular degeneration. Drugs Aging.

[18] Anguita R, Tasiopoulou A, Shahid S, Roth J, Sim SY, Patel PJ (2021). A review of aflibercept treatment for macular disease. Ophthalmol Ther.

[19] Eissing T, Stewart MW, Qian CX, Rittenhouse KD (2021). Durability of VEGF suppression with intravitreal aflibercept and brolucizumab: using pharmacokinetic modeling to understand clinical outcomes. Transl Vis Sci Technol.

[20] Sheen NJ, Fone D, Phillips CJ, Sparrow JM, Pointer JS, Wild JM (2009). Novel optometrist-led all Wales primary eye-care services: evaluation of a prospective case series. Br J Ophthalmol.

[21] Gágyor I, Hummers E, Schmiemann G, Friede T, Pfeiffer S, Afshar K, Bleidorn J (2021). Herbal treatment with uva ursi extract versus fosfomycin in women with uncomplicated urinary tract infection in primary care: a randomized controlled trial. Clin Microbiol Infect.

[22] Heier JS, Brown DM, Chong V (2012). Intravitreal aflibercept (VEGF trap-eye) in wet age-related macular degeneration. Ophthalmology.

[23] Wu J, He X, Qi F, Zhao Z, Xu Z, Yan H (2024). Efficacy, safety, and treatment burden of aflibercept 2 mg and ranibizumab in retinal vein occlusion: a systematic review and meta-analysis. Ophthalmol Ther.

[24] Carrasco J, Pietsch GA, Nicolas MP, Koerber C, Bennison C, Yoon J (2020). Real-world effectiveness and real-world cost-effectiveness of intravitreal aflibercept and intravitreal ranibizumab in neovascular age-related macular degeneration: systematic review and meta-analysis of real-world studies. Adv Ther.

[25] Chen Y, Wei W, Vavvas DG (2020). Incidence of endophthalmitis after intravitreal anti-vascular endothelial growth factor injections in an operating room in China. J Ophthalmol.

[26] Song W, Singh RP, Rachitskaya AV (2021). The effect of delay in care among patients requiring intravitreal injections. Ophthalmol Retina.

[27] Souied E, Nghiem-Buffet S, Leteneux C (2015). Ranibizumab prefilled syringes: benefits of reduced syringe preparation times and less complex preparation procedures. Eur J Ophthalmol.

[28] Russell MW, Chalasani M, Rana N (2023). Effect of prefilled vs vial-drawn syringes on sustained increases in intraocular pressure in patients treated with aflibercept. J Vitreoretin Dis.

